# FET PET to differentiate between post-treatment changes and recurrence in high-grade gliomas: a single center multidisciplinary clinic controlled study

**DOI:** 10.1007/s00234-024-03495-9

**Published:** 2024-11-11

**Authors:** Ameya D. Puranik, Indraja D. Dev, Venkatesh Rangarajan, Yash Jain, Sukriti Patra, Nilendu C. Purandare, Arpita Sahu, Amitkumar Choudhary, Kajari Bhattacharya, Tejpal Gupta, Abhishek Chatterjee, Archya Dasgupta, Aliasgar Moiyadi, Prakash Shetty, Vikas Singh, Epari Sridhar, Ayushi Sahay, Aekta Shah, Nandini Menon, Suchismita Ghosh, Sayak Choudhury, Sneha Shah, Archi Agrawal, N. Lakshminarayanan, Amit Kumar, Arjun Gopalakrishna

**Affiliations:** 1 Department of Nuclear Medicine and Molecular Imaging, Homi Bhabha National University, Tata Memorial Hospital, Mumbai, India; 2 Department of Radiodiagnosis, Tata Memorial Hospital and Advanced Center for Treatment, Research and Education in Cancer (ACTREC), Homi Bhabha National University, Mumbai, India; 3 Department of Radiation Oncology, Tata Memorial Hospital and Advanced Center for Treatment, Research and Education in Cancer (ACTREC), Homi Bhabha National University, Mumbai, India; 4 Department of Neurosurgery, Tata Memorial Hospital and Advanced Center for Treatment, Research and Education in Cancer (ACTREC), Homi Bhabha National University, Mumbai, India; 5 Department of Pathology, Tata Memorial Hospital and Advanced Center for Treatment, Research and Education in Cancer (ACTREC), Homi Bhabha National University, Mumbai, India; 6 Department of Medical Oncology, Tata Memorial Hospital and Advanced Center for Treatment, Research and Education in Cancer (ACTREC), Homi Bhabha National University, Mumbai, India; 7 Medical Cyclotron Facility, Board of Radiation and Isotope Technology (BRIT), Bhabha Atomic Research Center, Mumbai, India

**Keywords:** High grade glioma, FET PET, Recurrence, Post-treatment changes, MRI

## Abstract

**Purpose:**

The clinico-radiological dilemma in post-treatment high-grade gliomas, between disease recurrence (TR) and treatment-related changes (TRC), still persists. FET (Fluoro-ethyl-tyrosine) PET has been extensively used as problem-solving modality for cases where MR imaging is inconclusive. We incorporated a systematic imaging and clinical follow-up algorithm in a multi-disciplinary clinic (MDC) setting to analyse our cohort of FET PET in post-treatment gliomas.

**Methods:**

We retrospectively analyzed 171 patients of post-treatment grade III and IV glioma with equivocal findings on MRI. 185–222 MBq of 18 F-FET was injected and dedicated static imaging of brain was performed at 20 min. TBR (Tumor to background ratio) was used as semi-quantitative parameter. Cutoff of 2.5 was used for image interpretation. Imaging findings were confirmed with histopathological diagnosis, wherever available or in a multidisciplinary joint clinic based on serial imaging.

**Results:**

121 of 171 patients showed recurrent disease on FET PET, on follow up, 109 were confirmed with recurrence; 7 patients showed TRC, whereas 5 were treated with bevacizumab, with no further clinico-radiological deterioration, thus confirming TRC. 50 patients showed TRC on FET PET, on follow up on follow up, 40 were confirmed as true-negative. 10 patients who showed TBR less than 2.5 had confirmed TR on subsequent MR imaging. The overall sensitivity and specificity was 91.6 and 76.9% respectively, with a diagnostic accuracy of 87.13%.

**Conclusion:**

There is potential for FET PET to be used along with MRI in the post treatment algorithm of high-grade glial tumors.

## Introduction

Gliomas are the most commonly encountered primary malignant tumors of the central nervous system, arising from constituent glial cells of the brain parenchyma. The overall incidence is nearly 4.6 per 100,000 individuals per year and it has steadily increased over a past decade by 17.3% [1]. The treatment algorithms have evolved with the advent of better cross sectional imaging and molecular markers in pathology, however the overall 5-year survival rate remains poor with a range of 5% for glioblastoma (GBM) to 40% for low grade tumors, due to inherent aggressiveness and infiltrative nature of the tumor [2]. Neuroimaging remains the backbone in diagnosis and management of glial tumors, with MRI being the investigation of choice due to its high spatial resolution, better soft tissue demarcation and no exposure to ionizing radiation. Conventional sequences and modern techniques (like spectroscopy, diffusion-weighted and perfusion) of MR imaging have improved lesion characterisation and prognostication of brain tumors [3]. Considering the current treatment protocol for high grade gliomas, change in dynamics at the post-treatment site in the form of admixture of inflammation, neovascularisation and residual tumor poses a significant challenge in differentiating post-treatment changes from recurrent disease. Here, standard contrast-enhanced MRI provides low specificity [4]. Misinterpretation of treatment-related changes as progression may lead to a premature discontinuation of an ongoing effective treatment resulting in potentially negative impact on survival and an overestimation of the efficacy of the subsequent treatment [5]. The latter may also generate misleading results in studies evaluating salvage therapies. Amino acid PET tracers, like ^11^C-methionine (MET), ^18^F-fluoroethyl-L-tyrosine (FET), and fluorodopa (FDOPA), are transported via the L amino acid transporter type 1 (LAT1) system which is expressed on glial cells, and its expression is directly proportional to grade of glioma [6]. LAT1 is upregulated in cerebral gliomas, but the expression at the normal blood-brain barrier (BBB) is considerably lower [7]. Due to the fact that these amino acid tracers are also transported into the normal brain, disruption of the BBB is not a prerequisite for intratumoral localisation [8]. F-18 has a relatively long half-life of 110 min making 18F labeled amino acids as tracer of choice. FDOPA has normal localisation in the striatum, hence lesions in the vicinity can be obscured [9]. In addition, lesser cost of production and presence of in-house cyclotron made F18-FET (FET PET) the preferred tracer at our center. This retrospective study assessed a large patient cohort of high grade gliomas undergoing FET PET for differentiating post-treatment changes from recurrence. More importantly, each and every patient was systematically followed up on MRI and in neuro-oncology clinics, thereby ensuring a robust ‘gold standard’ to validate the results of FET PET.

## Materials and methods

This is a retrospective observational study approved by the Institutional Review Board and Ethics Committee (Project No 900717). 171 patients of World Health Organisation (WHO) Grade III and IV glioma, were studied. Patients first visited the Neuro-oncology out-patient department (OPD) following which, they underwent treatment as per institutional protocol for WHO Grade 3 and Grade 4 glial tumors. Patients subsequently were discussed in a joint neuro-oncology meeting (JNOM), which is an institutional multi-disciplinary clinic. FET PET was advised thereafter to differentiate between post treatment changes and recurrence. Patients referred for FET PET between 1st April 2017 and 30th April 2022, with a follow-up period of 18 months, were included in the study. Patient characteristics have been given in Table 1.

**Table 1:** Patient characteristics

Age (in years):	
18–55	45
Greater than 55	126
Gender:	
Male	116
Female	55
WHO Grade:	
Grade III Astrocytoma	59
Grade IV Glioblastoma	112
Follow up:	
MRI and MDC	139
Histopathology	18
FET PET, MRI and MDC	14

### Patient selection

Patients who were referred for FET PET after a multidisciplinary Joint Clinic decision were included in the study. These patients had a histopathological diagnosis of higher grade glioma (WHO Grade III or IV), and either underwent surgery followed by radiotherapy, or were deemed inoperable and received upfront radiotherapy. All patients received concurrent Temozolomide (TMZ) for 6 months, followed by adjuvant TMZ for 12 months, as the standard institutional protocol. All patients included in this study had a disease free period of at least 3 months following treatment completion and reported to OPD with clinical symptoms or equivocal MR findings. FET PET imaging was performed not later than 15 days from MRI. Following were the equivocal MRI findings which led to referral of patients for FET PET: (1) progressive or new contrast-enhancing lesion(s) on post contrast T1 or non-enhancing lesion(s) on T2/fluid-attenuated inversion recovery (FLAIR) MRI sequences, where the distinction between disease recurrence and posttreatment changes was uncertain, (2) irregular enhancement (‘Swiss Cheese’ pattern) typical for post-treatment changes in patients which showed areas of hyperperfusion and/or abnormal spectroscopy findings on MR imaging (3) patient showing clinical worsening with MR showing typical appearance of post-treatment changes. The above-mentioned referral pattern had 86, 48 and 37 patients in each subset, respectively.

### FET PET imaging protocol

Liesche et al. showed that dynamic PET parameters relied on tumor vascularity which was dependent on the extent of neovasuclarisation; and it was better depicted by MR perfusion imaging [10]. Static imaging at 20 min purely assesses the amino-acid receptor expression. In addition, a single-point imaging was justified in terms of scan time, costs, and patient turnover in a busy department. Patients were injected with 185–222 MBq of Fluorinated (Fluorine-18 labeled) Fluoro-ethyl tyrosine (FET). Dedicated static imaging of the brain was performed at 20 min post injection using Philips Gemini TF TOF 16/64 PET/CT scanners (PET crystal-LYSO). After obtaining a scout image, a plain and post contrast CT scan of the brain was performed in craniocaudal direction (120 kV, 250 mAs/slice, thickness-3 mm, increment − 1.5 mm, pitch of 0.9 and FOV of 300 mm). PET scanning was performed immediately after the CT acquisition in 3D mode, without changing the patient position. Single bed position was acquired, for duration of 5 min. Images were reconstructed iteratively using the row action maximum likelihood algorithm (RAMLA) algorithm. CT attenuation correction, dead time correction, and decay correction were applied.

### Image analysis

Reconstructed fused PET/CT images were viewed on Extended brilliance workspace (EBW) version 4.5, Philips Healthcare. Two independent Nuclear Medicine physicians, who were blinded to the MR findings, multi-disciplinary clinic discussions and histopathology; processed and reported the studies. Tumor to background ratio (TBR) was used as a semi quantitative parameter for image interpretation. It is defined as ratio of SUVmax of the lesion to the SUV mean of contralateral white matter. Background region-of-interest was based on recommendations in EANM, SNMMI, EANO, and RANO guidelines. A crescent-shaped volume of interest (VOI) was drawn in relation to healthy-appearing brain tissue in the hemisphere contralateral to the tumour including grey and white matter in the frontal lobe in six adjacent slices. A cut-off of 2.5 for tumor to white matter ratio was used to differentiate between tumor recurrence (TR) and treatment related changes (TRC). Positive FET study was suggestive of TR with a TBR of greater than 2.5, whereas negative FET study depicted TRC with TBR less than or equal to 2.5. Subsequently, the imaging findings were confirmed with either histopathological diagnosis in a multidisciplinary joint clinic or based on radiological or clinical follow up of patients.

### Clinico-radiological follow-up

The standard protocol for follow-up at our institution involves MR imaging and clinic visit at 6 and 12 months post-treatment completion, and annually thereafter. If the patient is symptomatic, the patient visits the clinic and MR is performed and discussed in a multidisciplinary clinic. In our study, following FET PET, patients underwent clinical follow-up and MR imaging every 3 months for the first 6 months and every 6 months thereafter. In all, 16 patients underwent surgery and histopathological confirmation was available.

### Statistical analysis

The diagnostic performance of the TBR for differentiation between TR and TRC was assessed by receiver-operating-characteristic curve analyses using the neuropathologic results or the clinicoradiological follow-up as a reference.

## Results

**Fig. 1:**
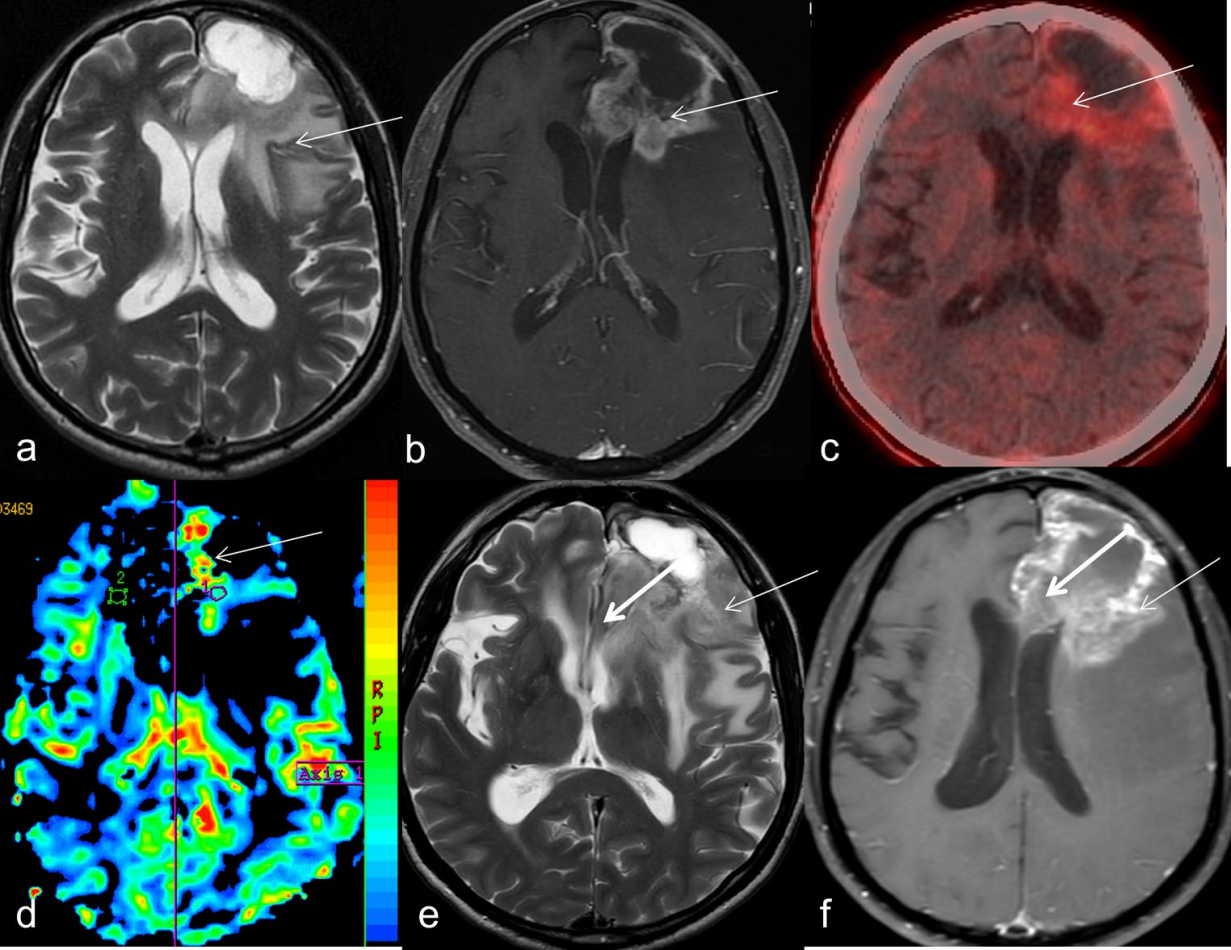
Disease recurrence on FET PET. 52-year-old male, case of anaplastic astrocytoma, post excision, received adjuvant radiotherapy along with temozolomide (TMZ), followed by 12# maintenance TMZ, had a disease-free interval of 14 months. T2-weighted axial image (A-arrow) on follow-up MRI showed iso-to-hypointense areas in left frontal region, seen as swiss-cheese pattern of enhancement on axial post-contrast T1-weighted image (B-arrow). Patient underwent FET PET for characterisation of lesion, which showed high T-Wm ratio of 3.6 suggestive of disease recurrence. Focal areas of hyperperfusion were seen (D-arrow). Since patient was well-preserved clinically, he was followed up after 3 months with MRI, which showed increase in extent of infiltration on T2-weighted (E- arrow) and post-contrast-T1 weighted image (F- arrow). MDC discussions corroborated the FET and follow up MR findings and concluded that patient had unequivocal disease recurrence and was started on salvage treatment

**Fig. 2:**
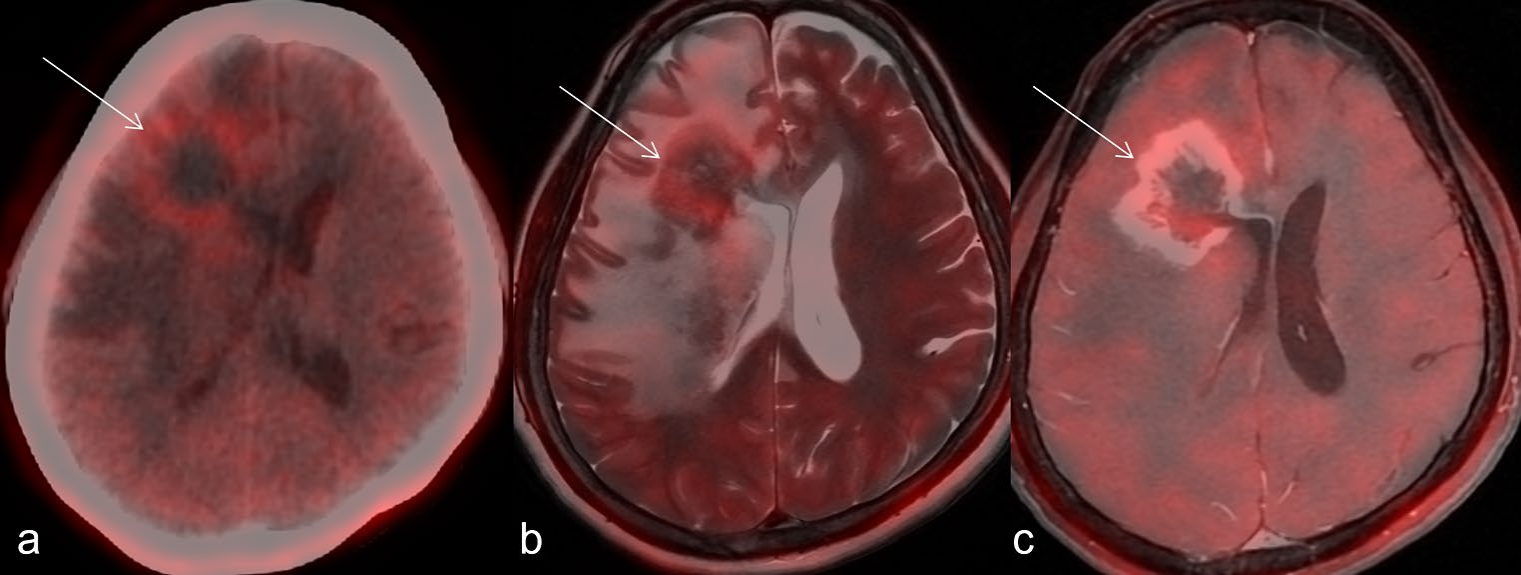
Post-treatment changes on FET PET. 39-year-old female, case of right parietal glioblastoma (GBM), post surgery, followed by concurrent radiotherapy-temozolomide (TMZ) and adjuvant TMZ, presented with left sided weakness, increased drowsiness. MR showed new onset T2 iso-to-hyperintense necrotic right periventricular lesion with post contrast irregular enhancement, and hyperperfusion in right temporoparietal region; FET PET showed low grade rim of tracer uptake in lesion (A - arrow), with tumor-to-white mater ratio of 1.8, suggestive of post-treatment changes. Fusion of PET and MR showed uptake corresponding to the rim of gliosis and enhancement on T2 (B - arrow) and T1-post contrast images (C - arrow). Patient was started on Bevacizumab following which improved symptomatically

Out of 171 patients who underwent FET PET (Table 2), 121 showed a TBR more than 2.5 and were reported as having recurrent disease. On follow up, 109 of them were confirmed as having recurrent disease (Fig. 1) based on either sequential imaging and/or histopathological diagnoses. Of the remaining 12 patients, MR of 7 patients was interpreted to have TRC, these patients were therefore observed and subsequent MR study (at 3 months) showed reduction in enhancement in the post-operative tumor bed, along with no clinical deterioration. 4 patients were treated with Bevacizumab and subsequent MR showed no change and there was no clinical deterioration over next 6 months, hence these were assumed to be TRC. In 1 patient there was no clinical worsening over thirteen months after which the patient showed unequivocal progression. Stereotactic biopsy was performed in 18 of these patients, whereas 14 patients underwent follow-up FET PET imaging. 50 patients were reported to have TRC, of which, on follow up, 40 were confirmed as true negative (Fig. 2). 10 patients who showed TBR less than 2.5 had confirmed TR on subsequent MR imaging (at 3 and 6 months from FET PET). The overall sensitivity and specificity was 91.6 and 76.9% percent respectively, with a diagnostic accuracy of 87.1% 9 (Table 2).

**Table 2:** Accuracy of FET PET findings in comparison with the final diagnosis

	Recurrence	Post-treatment changes	Total
**FET positive**	109	12	121
**FET negative**	10	40	50
**Total**	119	52	171

### Receiver operating curve analysis

**Fig. 3:**
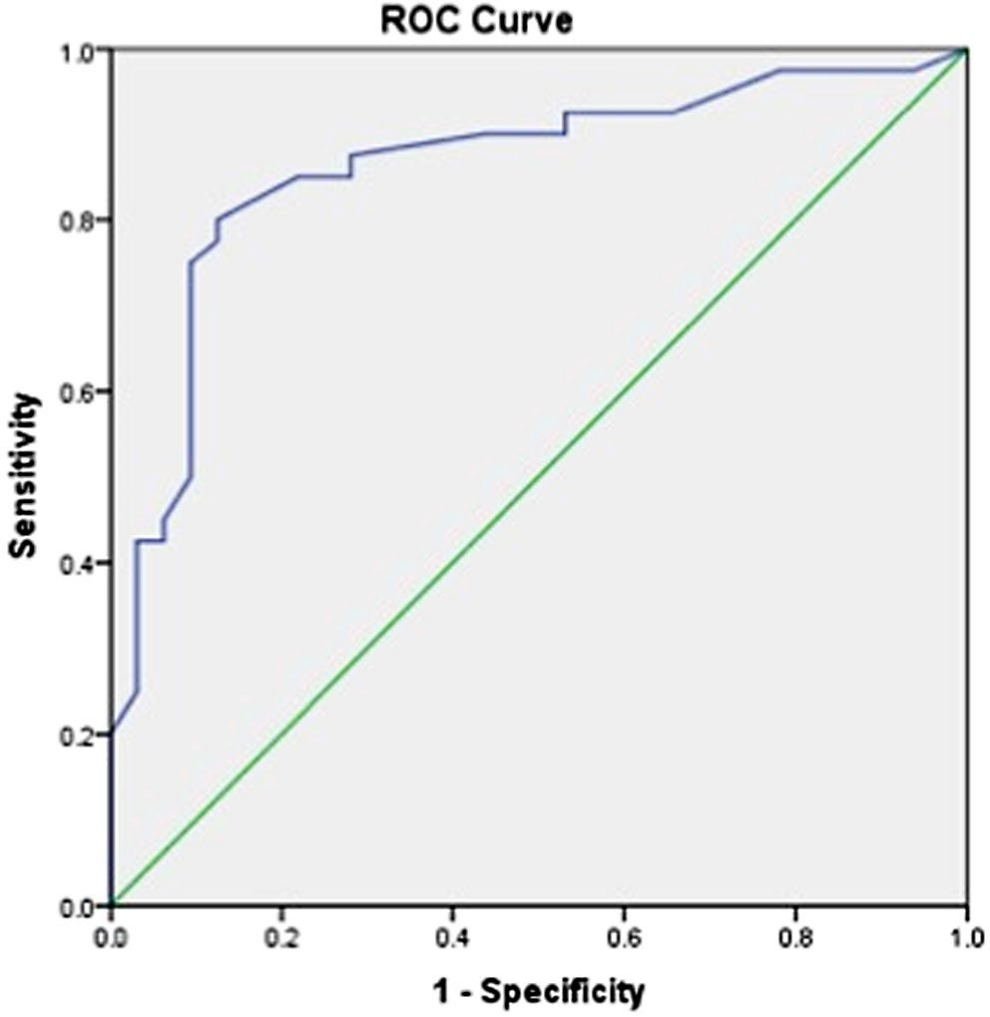
Receiver Operating Characteristics (ROC) curve for generating cut-off value for tumor-to-white mater ratio

ROC analysis (Fig. 3) was conducted in order to determine the cut off points of FET PET TBR. A statistically significant positive discrimination between true positive and true negative was revealed. Specifically, an excellent effect value was noted for FET PET TBR (AUC 0.992, *p* < 0.001). FET PET TBR cut-off point was equal to 2.65 with sensitivity of 94.4% and specificity of 100%.

## Discussion

Experimental and clinical studies have shown that FET uptake is highly specific for tumor tissue and is a result of increased LAT1 expression leading to a carrier-mediated facilitated transport in the glioblastoma tissue, and is thus independent of disruption of the BBB [11]. This makes FET an appropriate imaging modality for mapping proliferating glial tissue. Though MR imaging is multiparametric and has superior spatial resolution, it relies on tumor characteristics like infiltration, cellularity and neovascularization, which can also be seen in regions of post-treatment changes, affecting the overall sensitivity and specificity of the modality [12]. Perfusion-weighted MRI has additional parameters to overcome the shortcomings of conventional MRI [13]. Regional cerebral blood volume (rCBV) represents the most commonly employed and widely published metric for distinguishing TR from TRC [14]. However, there has been a wide variability in the reported rCBV thresholds across different studies, which ranged from 0.9 to 2.15 for mean rCBV [15]. Even RANO 2.0 is non-committal on utility of advanced imaging techniques to differentiate progression from pseudoprogression, and recommends further validation [16]. Functional imaging with amino acid tracers looks at the inherent proliferation of glial cells, which is tumor-specific. Since the onus is to identify active glial tissue in the background of reactive gliosis in post-treatment setting, it is important that we use a TBR cut-off which can reliably distinguish between high-grade glial tumor and non-tumor tissue. Hence, the cut-off of 2.5 was used in our study, which was derived by Rapp et al., with a high positive predictive value of 98% [17]. Our earlier study in 72 patients derived a TBR of 2.65 with a sensitivity of 80% and specificity of 87.5%. The current study with a larger sample size derived the same cut-off value [18], with a much higher sensitivity of 94.4% and specificity of 100%, respectively. This was primarily because of the learning that oligodendroglial tumors often lead to false positive FET uptake [19]; hence this histological subgroup was not included. There have been meta-analyses published assessing the diagnostic accuracy of PET tracers to distinguish between TR and TRC. de Zwart et al. included 10 studies on FET PET consisting of 201 patients yielding a pooled sensitivity and specificity of 90 and 86%, whereas Cui et al. included 15 studies to give a pooled sensitivity of 88 and 79% [20, 21]. The largest systematic review and meta-analysis on FET PET by Singnurkar et al. [22] included 26 studies on FET PET with 1209 patients. For static FET PET parameters, pooled sensitivity and specificity from six studies that expressed tumor uptake as TBRmax with a cut-off value of 1.9 to 3.69 were 91% and 84% respectively [23, 24]. This was in conjunction with the results of our study, which showed an overall sensitivity and specificity of 91.6 and 77% percent respectively. However these studies have predominantly relied on follow-up MR imaging as gold standard, whereas we used histopathology or follow-up FET PET in 32 patients. Since an equivocal MRI was the basis for referring patients for FET PET, it won’t be appropriate to rely on the same investigation alone for corroborating the FET results, hence an added discussion in MDC was done to give further validation. The MDC has access to FET findings at the time of discussion which may have caused a bias, however, it is important to understand that it is otherwise difficult to have ‘ground truth’ for patients with high-grade gliomas in post-treatment setting. This is because targeting a biopsy for histopathological diagnosis is challenging and biopsy specimens are seldom representative [25]. Also, periods of progression-free and overall survival are too short to study clinical impact of imaging findings. Treatment options like Bevacizumab are used for salvage therapy in recurrent GBM, and also prescribed for radiation necrosis in symptomatic patients; which again makes it difficult to validate the imaging results. These limitations can only be overcome by having a robust imaging and follow-up protocol, and by case-to-case discussion in a MDC [26]. The strength of our study is a large sample size, histopathological and FET PET correlation in nearly 20% patients, and a relatively long and standardized clinicoradiological follow up at specific time-points in an MDC setting. We would like to further strengthen our results by conducting the study in a prospective manner and also include PET volumetrics in the quantitation. In conclusion, we would like to propose that amino acid PET with FET PET should be incorporated in the post-treatment imaging algorithm of post-treatment high-grade glioma, as a complementary tool to MRI.

## Data Availability

In this manuscript there is no raw data such as nucleic acid sequences, protein sequences, genetic maps, etc. Nevertheless all the metaphases and picture are in possession of authors or available for reviewers or for submission in any database, if necessary.
